# The face of illusory truth: Repetition of information elicits affective facial reactions predicting judgments of truth

**DOI:** 10.3758/s13415-025-01266-4

**Published:** 2025-02-26

**Authors:** Annika Stump, Torsten Wüstenberg, Jeffrey N. Rouder, Andreas Voss

**Affiliations:** 1 Institute of Psychology, University of Freiburg, Freiburg im Breisgau, Germany; 2 Core Facility for Neuroscience of Self-Regulation (CNSR), Heidelberg University, Heidelberg, Germany; 3 University of California, Irvine, CA USA; 4 Institute of Psychology, Heidelberg University, Heidelberg, Germany

**Keywords:** Truth judgments, Truth effect, Affective states, Emotional expressions, Processing fluency, Retention interval length, Facial electromyography, EMG

## Abstract

People tend to judge repeated information as more likely true compared with new information. A key explanation for this phenomenon, called the illusory truth effect, is that repeated information can be processed more fluently, causing it to appear more familiar and trustworthy. To consider the function of time in investigating its underlying cognitive and affective mechanisms, our design comprised two retention intervals. Seventy-five participants rated the truth of new and repeated statements 10 min, as well as 1 week after first exposure while spontaneous facial expressions were assessed via electromyography. Our data demonstrate that repetition results not only in an increased probability of judging information as true (illusory truth effect) but also in specific facial reactions indicating increased positive affect, reduced mental effort, and increased familiarity (i.e., relaxations of musculus corrugator supercilii and frontalis) during the evaluation of information. The results moreover highlight the relevance of time: both the repetition-induced truth effect as well as EMG activities, indicating increased positive affect and reduced mental effort, decrease significantly after a longer interval.

People judge repeated statements as more familiar and trustworthy than novel statements even when the statements are the same (Dechêne et al., 2010; Hasher et al., 1977). This phenomenon—the illusory truth effect—appears quite robust and has been replicated in more than 80 published articles in the psychological literature after its first experimental demonstration by Hasher et al. (1977). The main goal of this paper is to establish an easy-to-detect physiological correlate of the illusory truth effect. For this purpose, we focus on specific facial expressions indicative of changes in fluency and affect and ask whether these are systematically related to the repetition of information and subsequent truth judgments.

The present literature suggests that the experience of high processing fluency caused by repetition is mainly responsible for the occurrence of the truth effect: People are using the subjective feeling of ease while processing information for judging its truthfulness (Dechêne et al., 2010). In fact, the relative processing fluency appears to be crucial. Dechêne et al. (2009) demonstrated that the effect only occurs when disfluent and fluent stimuli are presented intermixed. In truth effect experiments, fluency is typically manipulated via the presentation of novel and repeated information (e.g., information already presented in a prior phase, i.e., the exposure phase of the experiment). Garcia-Marques et al. (2016) argue that owing to a semantic and verbatim repetition, this manipulation should be considered as a source of both conceptual and perceptual processing fluency (see also Garcia-Marques et al., 2015). Former studies have shown that both the manipulation of conceptual fluency (e.g., Arkes et al., 1991) and perceptual fluency (e.g., Reber & Schwarz, 1999) can induce an illusory truth effect.

Another line of research demonstrated that not only processing fluency but also positive affective states are able to induce a feeling of familiarity and impact truth judgments. Garcia-Marques et al. (2004) showed that perceived positivity alone can signal familiarity (Exp. 2) and, furthermore, that a positive mood state can lead to more “true” judgments (neutral vs. positive mood state, Exp. 3). In fact, there also appears to be a link between perceived processing fluency and positive affect (Winkielman & Cacioppo, 2001; Winkielman et al., 2003; Topolinski et al., 2009).

Based on the present fluency and affect research, one could suggest that the truth effect, including the related processing fluency account, may also possess an essential affective component. Following this idea, Unkelbach et al. (2011) manipulated processing fluency via repetition as well as valence via the implementation of positive and negative information. Their results reveal that the valence of statements did not moderate the illusory truth effect. The authors concluded that positivity does not play a key role in the mechanisms underlying the truth effect but also pointed out that this interpretation should be seen as preliminary, because it relies entirely on null results (Unkelbach et al., 2011). More recent results suggest a link between affect, fluency, and illusory truth. Koch and Forgas (2012) induced mood states via film clips prior to their truth effect experiment. In their experiment, only perceptual processing fluency was manipulated; that is, statements were presented with varying degrees of readability. The authors observed a truth effect in the neutral but not in the negative mood condition. A decade later, Stump et al. (2022) reopened the question of whether affective changes triggered by experienced high versus low processing fluency play a crucial role in the mechanisms underlying the repetition-based truth effect. These authors manipulated affective states in a within-participant manner through affective priming and factorially crossed the affective manipulation with the repetition status of the statements (Stump et al., 2022, Exp. 1). The truth effect was reduced for statements that were presented with negative affective primes after a 1-week retention interval. Moreover, findings from a second experiment demonstrated that participants, who received an irrelevant source for potential changes in their affective experiences during the experimental sessions, showed a significantly diminished truth effect (Stump et al., 2022, Exp. 2). Taken together, these findings suggest that the repetition-based truth effect has an essential affective component and participants rely on their perceived affective states for judgments of truth when they do not attribute the current affective changes to an external (irrelevant) source. Furthermore, the results of Stump et al. (2022) show a substantial reduction of the truth effect after a longer retention interval and suggest that the affective mechanisms accompanying the fluency experience (caused by repetition) are likewise reduced as a function of retention interval length (subliminal negative affective primes reduced the truth effect after the longer 1-week but not after the short 10-minute interval, Exp. 1).

It is known that cognitive effort and affective states automatically evoke facial expressions, including specific facial muscle reactions, e.g., positive affect not only triggers smiling but also a relaxation of eyebrows (Ekman, 1973, 2003). Prior research shows that these specific facial muscle reactions can be measured by facial electromyography (fEMG)—even when the responses are subtle and fast (Dimberg et al., 2000). Three muscles in particular have been shown to be linked to experienced processing fluency and to be indicators for affective responses: *Musculus corrugator supercilii*, zygomaticus major, and frontalis. *Musculus corrugator supercilii* furrows the eyebrows. The muscle activity is known to be related to negative affective states (Cacioppo et al., 1986; Ekman, 2003) as well as mental effort (Cohen et al., 1992). *Musculus* *zygomaticus major*, which moves the corners of the mouth, has been shown to be associated with positive affect (Cacioppo et al., 1986; Ekman, 2003) as well as with increases of processing fluency (Harmon-Jones & Allen, 2001; Winkielman & Cacioppo, 2001). However, some results suggest that there is rather a quadratic relationship between valence and zygomatic tension (Lang et al., 1993) as well as findings that demonstrate a stronger linear effect for valence on muscle activity over the *M. corrugator* *supercilii* compared with *zygomaticus major* (Larsen et al., 2003). *M. frontalis*, which raises the brows, is an indicator for novelty and unexpectedness (Ekman, 2003; Scherer & Ellgring, 2007) but was also found to be associated with disfluency in the context of semantic coherence (Topolinski et al., 2009). Other studies that investigated the effects of surprising events in more ecologically valid ways, such as changing the whole room, revealed rather weak effects on frontalis responses (Reisenzein et al., 2006; Schützenwohl & Reisenzein, 2012).

In the present research, we implemented a classic repetition-based truth effect experiment, including two retention interval lengths (judgment phases: 10 min and 1 week after first exposure). Within the two judgment phases, participants had to judge different new and repeated statements to be true or false. At the same time, facial muscle reactions were measured using facial electromyography (fEMG) in three areas: *M. frontalis*, zygomaticus major, and corrugator supercilii. Based on the prior research, we hypothesized that information repetition (a) triggers specific facial muscle responses indicative of increased positive affect, experienced familiarity and decreased mental effort, and (b) increases the likelihood of judging statements as true. We further expected a time-related decrease of the truth effect, including an accompanying reduction of the specific muscle responses caused by repetition.

## Method

We analyzed data from a larger study, which was designed to address several research questions. Informed consent was obtained from all study participants. This study was approved by the ethics committee of Heidelberg University (Faculty of Behavioural and Cultural Studies) and was conducted in accordance with the Declaration of Helsinki. In the following, we describe all procedures and measures that are relevant to the present hypotheses.

### Participants and design

In total, 81 student participants were recruited at Heidelberg University with the recruitment software hRoot (Bock et al., 2014). We excluded data from three participants who did not attend the second testing session, from two participants who aborted the experiment, and from one person because of a technical error. The remaining 75 participants were between 18 and 31 years old (*M* = 21.63, *SD* = 2.96), and the majority of the sample (81%) was female (13 participants indicated male and 1 person indicated diverse as gender).^1^ The majority of participants (68%) were non-psychology students. Participants received 15 Euros (approximately 16 US$) or course credit for their participation.

The study design comprised the two within-subject factors *repetition status* (new vs. repeated) and *retention interval* (10 min vs. 1 week).

### Material

The main statement material included 120 statements in German language (4 statement sets). Additionally, 12 statements were selected to counteract possible primacy as well as recency effects (first and last 6 statements during the exposition phase). Unkelbach and Stahl (2009) demonstrated that people use their fluency experiences, especially for judgments of truth when there is relatively high uncertainty about the factual truth of a statement. We therefore selected statements by Nadarevic (2010) that had been carefully tested to be difficult enough for most participants and produced a reliable illusory truth effect in former studies (Nadarevic, 2010). Exemplary statements are “An ostrich's eyes are bigger than its brain.” (factually correct) or “A queen bee lays an average of 500 eggs a day.” (factually incorrect)—note that these are English translations of the original German statements. Furthermore, we only used statements that we perceived as being affectively neutral, as their content should not induce any affective reactions. We also ensured that all statements were about the same length so that the individual processing of them would take a similar amount of time. The statements were divided into four sets, including 15 true and 15 false statements. We ensured that truth ratings (as indicated by Nadarevic, 2010) had comparable means as well as standard deviations in all statement sets (set A: *M* = 4.06, *SD* = 1.20; set B: *M* = 4.06, *SD* = 1.18; set C: *M* = 4.05, *SD* = 1.18; set D: *M* = 4.05, *SD* = 1.20; statements were rated on a seven-point scale). In every judgment phase, two statement sets were used, whereby one set in each judgment phase was already presented during the exposure phase. The assignment of the different statements sets to the experimental phases was counterbalanced across subjects.

### Procedure

After receiving the consent form and the subsequent placement of the EMG electrodes on the participants’ faces, the computer experiment started. During the first experimental phase, 72 statements were presented trial-by-trial. Participants had to classify the statements into specified categories: (1) Geography, (2) Biology, (3) Politics & History, (4) Science, (5) Entertainment, and (6) Others. Six statements at the beginning and at the end of this task served as a buffer against potential primacy as well as recency effects and therefore remained the same for all participants. The 60 statements in between were taken from two statement sets (including 15 true and 15 false statements each) and shown in random order. Every trial started with a fixation cross for 1000 ms, followed by one statement presented in the center of the screen until the participants did their classification by key press. Participants were able to classify the presented statement into one of the six knowledge categories by pressing one of the number keys (1 to 6). There was no time limit for this task; however, participants were instructed to respond as quickly as possible while avoiding unnecessary mistakes.

In a subsequent 10-minute retention interval, the subjects worked on a non-verbal filler task; thereafter, the first judgment phase of the experiment started. During the judgment phase, 30 novel and 30 repeated statements were shown trial-by-trial in random order. The 30 new statements were taken from one of the statement sets not used so far, whereas 30 of the statements (repeated statements) originated from one of the sets presented during the exposure phase. Each trial started again with a fixation cross that was displayed for 1000 ms. The subsequently presented statement remained on the screen until participants gave their truth judgment by pressing the “W” key (“wahr”; German for true) or “F” key (“falsch”; German for false). Afterwards, participants rated the confidence with which their previous truth judgment was made by pressing one of the upper number keys (1 “very uncertain” to 6 “very certain”). There was no time limit for judgments. However, as in the exposition phase, subjects were instructed to make their judgments as quickly as possible, without avoidable mistakes.

In the second experimental session, which was conducted exactly 7 days after the first one, subjects attended the second judgment phase. The procedure was similar to the one in the first judgment phase, except that new material was used. That is, statements from the final unused statement set were presented intermixed with those statements from the exposition phase, which had not been presented during the first judgment phase.

We explicitly informed our participants prior to all experimental phases that both true and false statements would be presented.

#### EMG assessment

Electrical activities of facial muscles were sampled at 1 kHz and 16 bit over left *M. corrugator supercilii, M. zygomaticus major,* and *M. frontalis* using pre-gelled adhesive Ag/AgClsurface-electrodes (Mini Adhesive Electrodes, Kendall™ H124SG). Recordings were taken using a wireless Biosignals PLUX 8-channel hub and transferred in real-time to an acquisition computer via Bluetooth. Data was filtered with a 4th order band-pass filter (10 Hz low cutoff, 400 Hz high cutoff) and a 50 Hz notch filter. After rectification, the amplitude envelope was computed using a moving average of 30 ms width.

**Fig. 1:**
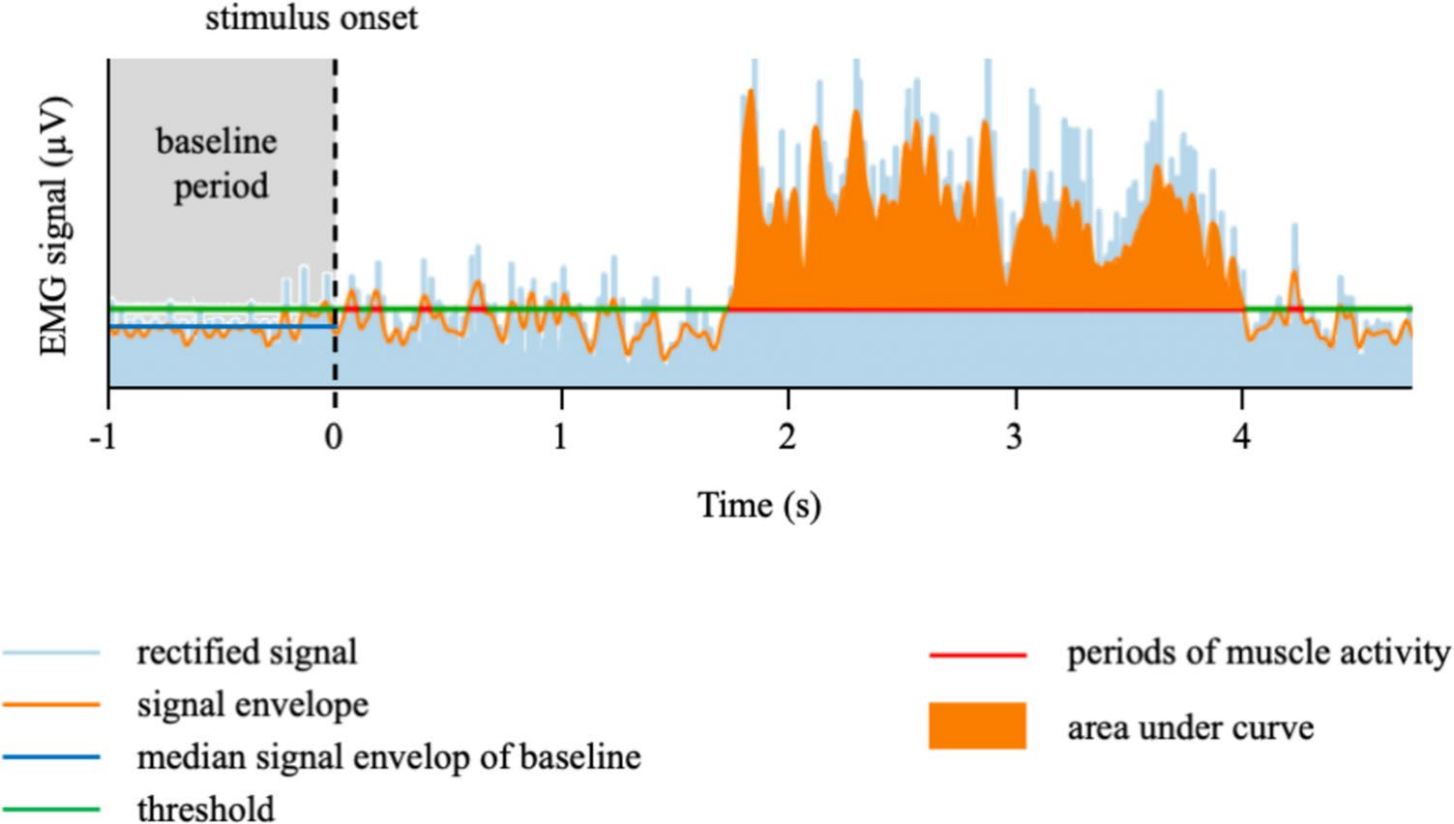
*Note.* Schematic depiction of the extraction approach, illustrating the segmentation and subsequent extraction of muscle activities

For muscle activity detection, envelopes were segmented based on experimental stimulation and participant response. With the aid of light (LUX) sensors the start and end points of all trials were identified. That is, specific bright rectangles appeared under the LUX sensors in the corners of the computer screen at the time of stimulus onset and keypress by participants (not visible for subjects).^2^ To consider signal noise and pre-stimulus muscle activity, the activity detection threshold was estimated trial-wise using the median plus one standard deviation of the baseline envelope (baseline period = 1000 ms before each statement presentation). All sampling points with an envelope above this threshold and a minimum duration of 50 ms were considered as active. Based on these criteria, we extracted for each trial the area under the curve of the rectified EMG signal for each muscle area (see Fig. 1 for a schematic depiction of the extraction approach). To consider the variation in trial durations the areas under curves (AUC) were relativized to the response times (RT) for each trial (AUC/RT).

## Results

A multilevel modeling approach was used for all analyses to account for the hierarchical structure of the data. The analyses were performed with the statistical software R (version 4.3.3) using the lme4 package (Bates et al., 2015) in combination with the lmerTest package (Kuznetsova et al., 2017) for calculating *p* values. A linear mixed model fit by maximum likelihood was used. All models included random intercepts for subjects.^3^ For the analyses, the level-1 predictor *repetition status* was dummy coded, whereby the new statements served as reference condition.

**Fig. 2:**
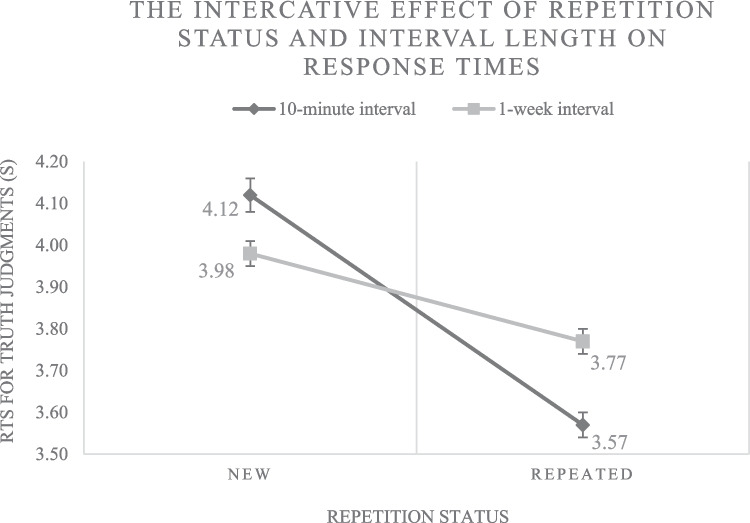
*Note.* The mean reaction times for truth judgments of new (disfluent) and repeated (fluent) statements as a function of interval length between presentations (repetition after a retention interval of 10 min vs. 1 week). Error bars represent standard errors

As a manipulation check, we analyzed whether repetition (considered to be a source of conceptual as well as perceptual processing fluency) influenced the response times for judgments of truth.^4^,^5^ Additionally, we checked for potential effects related to the retention interval length and veridicality of the statements presented. For this purpose, the predictors *repetition status*, *judgment phase* (coded −0.5 for the first and +0.5 for the second judgment phase), *veridicality* (coded −0.5 for factually incorrect and +0.5 for factually correct statements) as well as interactions between *repetition status* and (i) *judgment phase* as well as (ii) *veridicality* were implemented in the model. Results demonstrated a significant main effect for repetition status (*b* = −.106, *p* < .001) and judgment phase (*b* = −.040, *p* < .001), as well as an interaction between repetition status and judgment phase (*b* = .103, *p* < .001). No significant effects related to the veridicality of the statements were observed (veridicality: *b* = −.002, *p* = .846; repetition status x veridicality: *b* = −.001, *p* = .918). Our results indicate that participants made their truth judgments faster when a statement was repeated. Notably, the time between first exposure and the later judgment phases significantly influenced the effect of repetition status, indicating that the magnitude of the repetition-based fluency manipulation decreases with rising retention interval length (see Fig. 2 for an illustration of the measured response times underlying this result).

To account for the impact of interval length and for the sake of a better comparability with other research (given that the majority of truth effect studies include only one interval length), the subsequent models to predict (i) facial EMG responses and (ii) truth judgments were performed for the first and second judgment phase separately. Prior to these separate analyses, we will report our direct tests with respect to the potentially moderating effects of the retention interval length and potentially confounding effects of veridicality.

### EMG responses

For analyses of the EMG responses in the different facial parts, we used the areas under curves divided by the response times per trial. We added a constant (+1.0) to the relativized AUC (in microvolt) and then logarithmized the data. Subsequently, we removed extreme values (defined as lying more than 3.0 interquartile ranges below the first or above the third quartile of the logarithmized distributions) prior to the respective analyses.

### Linked indicators of fluency: EMG activities and reaction times

As described in the introduction, previous research has already linked activity in all three muscles (corrugator supercilii, frontalis, and zygomaticus major) to processing fluency (e.g., Topolinski et al., 2009). Since reaction times are also considered a common indicator of experienced fluency, we tested, following the suggestion of a reviewer, to what extent the activities in the different facial muscle areas are related to the speed of judgments. For this purpose, we included corrugator supercilii, frontalis, and zygomaticus major activities as predictors in a model analyzing the response times per trial. The results revealed significant positive main effects for all facial muscle parts (stronger EMG activities were associated with slower judgments), whereby the corrugator activities appear to be most strongly associated with the judgment times (corrugator supercilii: *b* = .035, *p* < .001; frontalis: *b* = .012, *p* < .001; and zygomaticus major: *b* = .018, *p* < .001).

### Repetition-based EMG responses as a function of retention interval length

Prior to the analyses which were conducted separately for the first and second judgment phases, we directly tested whether the retention interval significantly influences repetition effects on the corrugator supercilii, frontalis, and zygomaticus major activities. Additionally, we checked for potentially confounding effects related to the veridicality of the statements presented. Consequently, we included the predictors *repetition status*, *judgment phase* (coded −0.5 for the first and +0.5 for the second judgment phase), *veridicality* (coded −0.5 for factually incorrect and +0.5 for factually correct statements) as well as interactions between *repetition status* and (i) *veridicality* as well as (ii) *judgment phase*.

The results of our first model, which was built to predict *corrugator activities*, revealed a significant main effect of repetition status (*b* = −.114, *p* < .001) and judgment phase (*b* = −.265, *p* < .001), as well as an interaction between repetition status and judgment phase (*b* = .238, *p* < .001). These findings indicate that participants showed reduced *corrugator tensions* when statements were repeated. Moreover, the retention interval length significantly moderated the effect of information repetition on corrugator activities; that is, the effect of repetition on *corrugator activities* was markedly reduced after the longer retention interval (1 week vs. 10 min). No significant effects related to the veridicality of the statements were observed (veridicality: *b* = −.015, *p* = .696; repetition status x veridicality: *b* = −.003, *p* = .962).

In the following model, we included the same predictors (as described above) to predict activities of *zygomaticus major*. The results revealed only a negative main effect of veridicality (*b* = −.093, *p* = .033), indicating reduced zygomaticus tensions when factually correct (vs. incorrect) statements were presented. No other significant main or interaction effects were found (repetition status: *b* = −.060, *p* = .051; judgment phase: *b* = −.063, *p* = .147; repetition status x judgment phase: *b* = .041, *p* = .507; repetition status x veridicality: *b* = −.008, *p* = .891).

Finally, we included the above-described predictors in a third model to predict *frontalis activities*. Results revealed a significant main effect of repetition status (*b* = −.090, *p* = .002) and judgment phase (*b* = −.084, *p* = .046), indicating increased frontalis activities (i) when statements were presented for the first time (vs. repeatedly), and (ii) after a shorter (10 min vs. 1 week) retention interval (in the case of new statements). No other significant main or interaction effects were observed (veridicality: *b* = −.071, *p* = .088; repetition status x veridicality: *b* = .011, *p* = .853; repetition status x judgment phase: *b* = .090, *p* = .128).

### EMG responses after the 10-minute interval

**Fig. 3:**
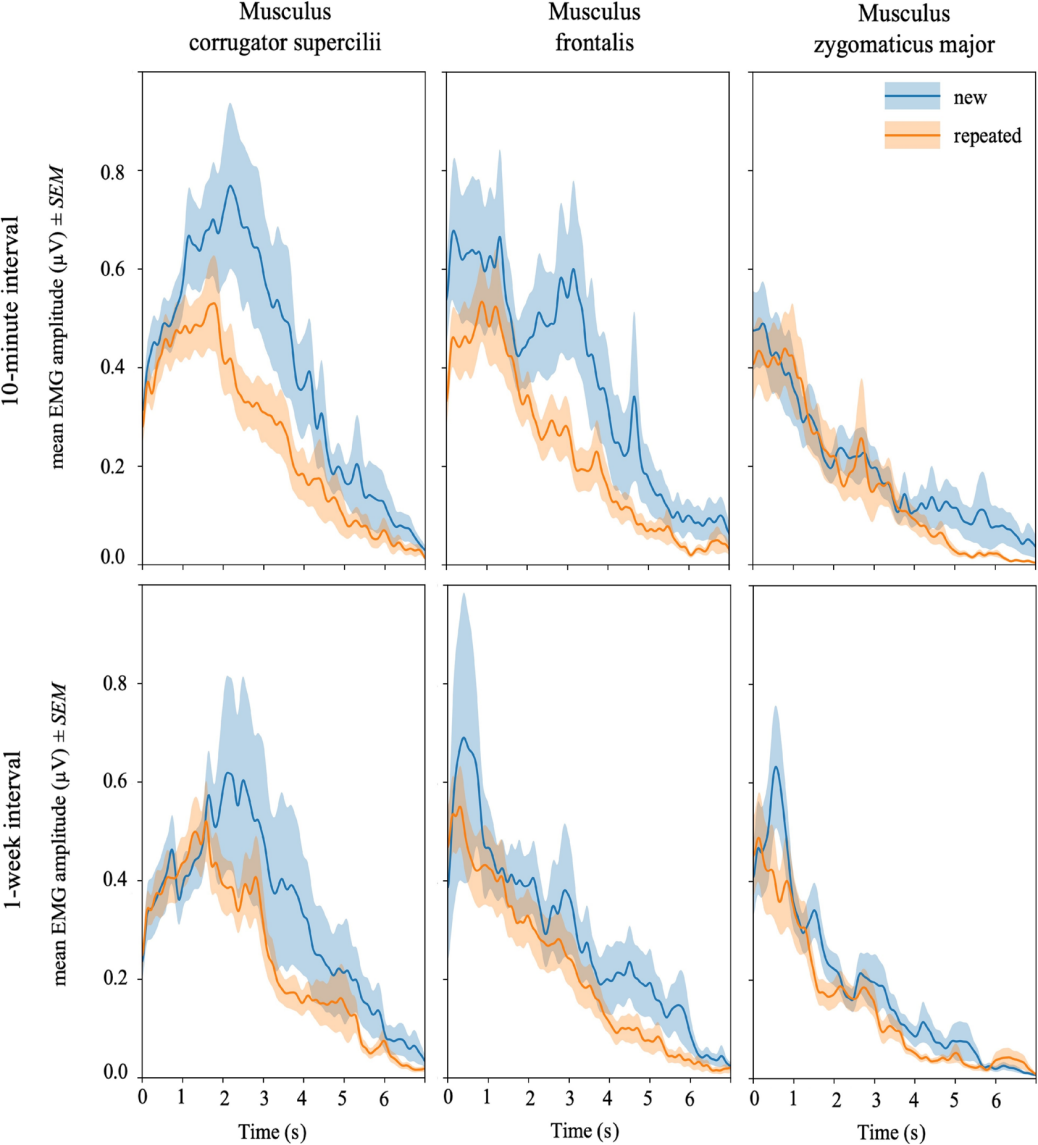
*Note.* The mean temporal dynamics of musculus corrugator supercilii, frontalis, and zygomaticus major for presented new (disfluent) and repeated (fluent) statements until participants gave their truth judgments via keypress during judgment phases 10 min versus 1 week after first presentation. For this purpose, the sampling point wise mean values were extracted for all activity phases of all participants. Then, the mean activity curves and variations were calculated for new and repeated statements separately. Shaded regions represent standard errors

Again, activities of corrugator supercilii, frontalis, and zygomaticus major as a function of information repetition were analyzed in separate multilevel models (see Table 1 for all results and Fig. 3 for an illustration of the EMG activities underlying all results).

**Table 1:** Multilevel modelling results for the prediction of EMG responses (i.e., logarithmized AUC)

Fixed effects								
		10-min interval			1-week interval			
	*b*	*SE*	*t*	*p*	*b*	*SE*	*t*	*p*
*Log. Corrugator Activity*								
Intercept	2.797	.093	30.095	<.001***	2.533	.089	28.350	<.001***
Repetition status	−.233	.039	−6.044	<.001***	.006	.037	.170	.865
*Log. Frontalis Activity*								
Intercept	2.318	.067	34.769	<.001***	2.234	.063	35.269	<.001***
Repetition status	−.136	.042	−3.239	.001**	−.044	.041	−1.091	.276
*Log. Zygomaticus Activity*								
Intercept	1.809	.053	34.440	<.001***	1.743	.049	35.439	<.001***
Repetition status	−.081	.044	−1.852	.064	−.040	.042	−.939	.348

*Notes. N* = 75. ***p < .001; **p < .01

In a first model, the level-1 predictor repetition status was included to predict the logarithmized *corrugator* activity (as an indicator of changes in mental effort and affect). In line with our hypotheses, results reveal a negative main effect for repetition status (*b* = −.233, *p* < .001), implying significantly decreased corrugator activities for repeated compared to new statements. This finding indicates increased positive affect and reduced mental effort during the information processing of repeated (vs. novel) statements. However, since repetition influences the perception of truth, and this may also be related to increased positive affect, we conducted an additional analysis. To test whether activity changes in corrugator activities during information processing are triggered by the repetition status—beyond related truth impressions—we included, in addition to the predictor repetition status in Model 1, the judgment (coded 0 for “false” and 1 for “true” responses) and an interaction involving both variables. Results revealed main effects for both repetition status and truth judgment, implying that repetition has an impact on corrugator activities over and above a response-related effect (repetition status: *b* = −.209, *p* = .002; truth judgment: *b* = −.255, *p* < .001; repetition status x truth judgment: *b* = .037, *p* = .656).^6^ This finding is further supported by an inspection of the temporal dynamics of muscle activities shown in Fig. 3: Differences in corrugator activities can be seen very early (0–2000 ms after the start of statement presentations in session 1); at this early stage of information processing effects of the later response “true” vs. “false” seem rather unlikely.

In the following model, the predictor repetition status was included to predict the logarithmized *frontalis* activity (as an indicator of experienced novelty). Results show again a negative main effect for repetition status (*b* = −.136, *p* = .001), implying significantly reduced frontalis activities, indicating less experienced novelty, when processing repeated in comparison to new statements.

In a third model, the predictor repetition status was integrated to predict the logarithmized *zygomaticus* activity (as an indicator of changes in affect). Results reveal a marginally significant negative main effect of repetition status (*b* = −.081, *p* = .064), suggesting slightly reduced zygomaticus activities for repeated statements. A repetition-related reduction in zygomaticus activity (often referred to as the “smiling muscle”) may be unexpected to some readers. Regarding this concern, we would like to point out once again that there are prior results suggesting a quadratic relation between valence and zygomatic tension (Lang et al., 1993), as well as findings that demonstrate a stronger linear effect for valence on muscle activity over the musculus corrugator supercilii in comparison to zygomaticus major (Larsen et al., 2003). Our empirical data support these findings. In a further analysis, we integrated corrugator responses in addition to the predictor repetition status into the model to predict the zygomaticus activities. Results revealed a significant positive effect of corrugator activity on zygomaticus activity (*b* = .079, *p* < .001), whereas the previous found marginally significant effect of repetition status was further reduced (*b* = −.063, *p* = .154). These findings indicate that the present zygomaticus activities are associated with eyebrow frowning, suggesting facial expressions indicating rather increased negative affect when processing new compared to repeated information.

### EMG responses after the 1-week interval

As for the data of the first judgment phase (collected after a 10-minute interval), we built separate models to predict the activities in the different facial areas (i.e., for the muscles corrugator supercilii, frontalis, and zygomaticus major). The analyses reveal no significant impact of repetition status on the facial muscle activities in all three areas (corrugator supercilii: *b* = .006, *p* = .865; frontalis: *b* = −.044, *p* = .276; zygomaticus major: *b* = −.040, *p* = .348), suggesting similar facial reactions for new versus repeated stimuli after the 1-week interval. These findings further highlight the significance of time regarding effects of information repetition and are well in line with the expected reduction in repetition-induced affective changes as a function of time. Table 1 displays all results. For an illustration of the EMG activities underlying all results, see Fig. 3.

### Truth judgments

Truth judgments were analyzed using a generalized linear mixed model basing on maximum likelihood (Laplace approximation). To account for the dichotomy of the judgments (true vs. false), a logit link function was used.

### The truth effect as a function of retention interval length

Prior to the analyses conducted for the first and second judgment phase separately, we directly tested whether the retention interval between the first exposure and later judgment phases significantly influenced effects of repetition and facial expressions on truth judgments. Additionally, we checked for potentially confounding effects related to the veridicality of the statements presented. Consequently, the predictors *repetition status*, *judgment phase* (coded −0.5 for the first and +0.5 for the second judgment phase), *veridicality* (coded −0.5 for factually incorrect and +0.5 for factually correct statements), the logarithmized *corrugator activity*, logarithmized *frontalis activity*, and logarithmized *zygomaticus activity* were implemented in the model. Furthermore, we included interactions between *judgment phase* and (i) *repetition status*, (ii) *log. corrugator* *activity*, (iii) *log. frontalis* *activity*, and (iv)* log. zygomaticus activity* as well as between *veridicality* and (i) *repetition status*, (ii) *log. corrugator* *activity*, (iii) *log. frontalis* *activity*, and (iv) *log. zygomaticus* *activity*. Our results revealed significant main effects for repetition status (*b* = .609, *p* < .001), corrugator activities (*b* = −.129, *p* < .001), and veridicality (*b* = −.236, *p* = .049). These findings indicate an increased probability of “true” (vs. “false”) judgments (i) for repeated (vs. new) statements, (ii) when participants showed decreased corrugator activities, and (iii) for factually incorrect statements.^7^ Furthermore, we found a pronounced interaction effect between repetition status and judgment phase (*b* = −.566, *p* < .001), indicating that an increasing time interval between first exposure and later judgment phase significantly reduced the illusory truth effect. The veridicality of the statements did not influence any of the other effects investigated (veridicality x repetition status:* b* = .077, *p* = .393; veridicality x log. corrugator activity: *b* = .017, *p* = .595; veridicality x log. zygomaticus activity: *b* = −.022, *p* = .492; veridicality x log. frontalis activity: *b* = .025, *p* = .442). No significant interaction effects between *judgment phase* and (i) *log.* *corrugator* *activity* (*b* = −.004, *p* = .906), (ii) *log.* *frontalis* *activity* (*b* = .002, *p* = .957), and (iii*) log.* *zygomaticus activity* (*b* = .042, *p* = .183) were observed.

### Truth judgments after the 10-minute interval

In a first model, the level-1 predictor *repetition status* was included. Results demonstrate a significant main effect (*b* = .924, *p* < .001), indicating an increased probability of truth ratings for repeated in comparison to new statements. The odds ratio (OR) is 2.52, indicating that the judgment "true" (vs. "false") was 2.52 times more likely when a statement was repeated. Thus, we replicated the basic truth effect: individuals are more likely to judge repeated (fluent) information as true compared with new (disfluent) information. In a second model, the predictors *repetition status*, the *logarithmized corrugator activity*, *logarithmized frontalis activity*, and *logarithmized zygomaticus activity* were integrated. In addition to the main effect of *repetition status* (*b* = .899, *p* < .001; OR = 2.46), the predicted negative main effect for the *corrugator activity* was revealed (*b* = −.135, *p* < .001; OR = .87). The negative value of the regression weight implies a decreased probability for “true” judgments when corrugator activities were higher (which are indicative for increased negative affect as well as mental effort). Main effects for the *zygomaticus activity* (*b* = −.017, *p* = .454) and *frontalis activity* (*b* = .010 *p* = .697) were not significant*.* Table 2 shows all results. To test whether the effect of repetition status on truth judgments was (partially) mediated by corrugator activity (indicative for changes in fluency and affect), we additionally performed a mediation analysis using the lme4 package (Bates et al., 2015) in combination with the mediation package (Tingley et al., 2014). With the aid of bootstrapping procedures (1000 simulations), the analysis revealed the significance (*p* < .001) of the partial mediation effect, which was already shown descriptively in the prior pattern of results.

**Table 2:** Multilevel logistic modelling results for the prediction of "true" responses

Fixed effects								
		10-min interval				1-week interval		
	*b*	*SE*	*z*	*p*	*b*	*SE*	*z*	*p*
*Model 1*								
Intercept	.154	.062	2.472	.013*	.272	.055	4.936	<.001***
Repetition atatus	.924	.066	14.038	<.001***	.320	.062	5.138	<.001***
*Model 2*								
Intercept	.542	.105	5.178	<.001***	.489	.092	5.291	<.001***
Repetition status	.899	.066	13.596	<.001***	.325	.063	5.190	<.001***
Log. Corrugator activity	−.135	.025	−5.317	<.001***	−.121	.025	−4.859	<.001***
Log. Frontalis activity	.010	.024	.390	.697	.019	.024	.770	.442
Log. Zygomaticus activity	−.017	.023	−.749	.454	.029	.023	1.287	.198

*Notes. N* = 75. ***p < .001; *p < .05

A decrease in the Akaike information criterion (AIC) and Bayesian information criterion (BIC) indicated an improved model fit for the model including EMG predictors (AIC = 5506.2; BIC = 5544.6) compared with the more parsimonious model, including only repetition status as predictor (AIC = 5531.3; BIC = 5550.5), suggesting that the facial EMG data explained substantial variance in truth judgments.

### Truth judgments after the 1-week interval

**Fig. 4:**
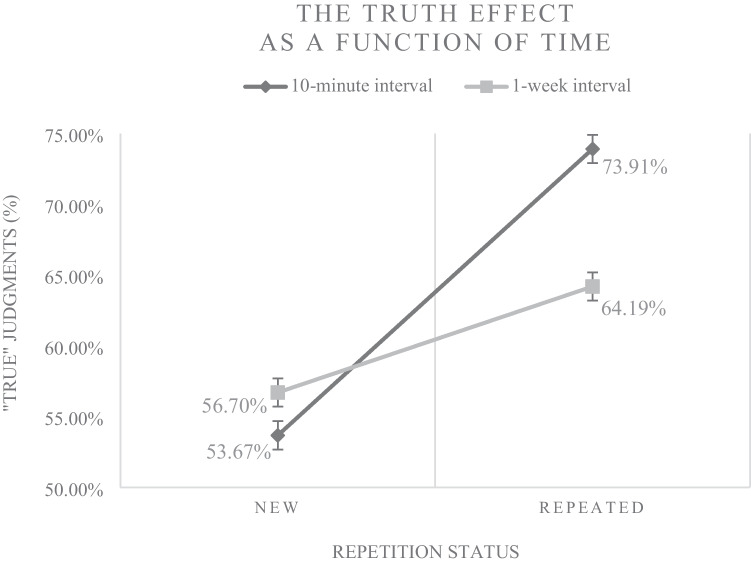
*Note.* The percentages of new (disfluent) and repeated (fluent) statements judged true by participants 10 min versus 1 week after first exposure. Error bars represent standard errors

Again, the level-1 predictor *repetition status* was included in a first model. A repetition-based truth effect was also found after the 1-week interval (*b* = .320, *p* < .001; OR = 1.38), indicating that the response “true” (vs. “false”) was 1.38 times more likely when a statement was presented repeatedly. Compared with the short (10-minute) interval, a substantial decrease in the effect size can be noticed (main effect of *repetition status* after the 10-minute interval: *b* = .924, *p* < .001; OR = 2.52). Figure 4 illustrates the observed frequencies underlying the truth effects after the short (10-minute) versus the longer (1-week) interval.

In a next step, the predictors *repetition status*, the *logarithmized corrugator activity*, *logarithmized frontalis activity*, and *logarithmized zygomaticus activity* were included in Model 2. In addition to the main effect of repetition status (*b* = .325, *p* < .001; OR = 1.38), and similar to the short (10-minute) interval, a negative main effect of the corrugator activity was found (*b* = −.121, *p* < .001; OR = .89), implying a decreased probability for “true” judgments when corrugator activities were higher. Main effects of the *zygomaticus activity* (*b* = .029, *p* = .198) and *frontalis activity* (*b* = .019, *p* = .442) were not significant. Table 2 displays all results.

AIC and BIC indicated an improved model fit for the second (larger model) compared with the first model (Model 1: AIC = 5896.4 & BIC = 5915.6; Model 2: AIC = 5877.0 & BIC = 5915.4), suggesting that the facial EMG data explained substantial variance in truth judgments also after the relatively long (1-week) retention interval.

## Discussion

The central aim of the present research was to investigate an easy-to-detect physiological correlate of the repetition-induced truth effect. To this end, we focused on facial expressions indicative of changes in fluency and affect. Earlier research suggests that the time between first exposure and later repetition is a key moderator in this context (Stump et al., 2022). Therefore, we not only conducted a repetition-based truth effect experiment and assessed facial EMG responses, but we also systematically manipulated the retention interval length within participants. That is, we implemented two judgment phases: (a) 10 min and (b) 1 week after first exposure.

With our present study, we replicated the repetition-based truth effect as well as the significant impact of time on its effect size. Individuals were more likely to judge repeated statements as true in comparison to statements presented for the first time. As in previous studies, this illusory truth effect was reduced with rising retention interval length (10 min vs. 1 week after first exposure). The significance of time was reflected not only in the pure size of the truth effect, but also in the repetition-induced muscle responses indicative for cognitive and affective processes and the repetition-related judgmental speed.

After the 10-minute interval the presentation of repeated information, which had to be judged as being true or false, resulted in (a) reduced activities of *corrugator* *supercilii* indicating increased positive affect (Cacioppo et al., 1986; Ekman, 2003) and decreased mental effort (Cohen et al., 1992), in (b) a reduction of *frontalis* activities indicating increased familiarity (Scherer & Ellgring, 2007), as well as in (c) marginally reduced activities of *zygomaticus* *major*. The marginally repetition-related decrease in activity of the zygomaticus muscle (commonly known as the “smiling muscle”) might be surprising to some readers. However, it is important to note that previous research indicates a rather quadratic relationship between valence and tension in the zygomaticus muscle (Lang et al., 1993), as well as a stronger linear effect for valence on muscle activity in the musculus corrugator supercilii (which is responsible for eyebrow frowning) compared with zygomaticus major (Larsen et al., 2003). Our empirical data support these findings, suggesting that corrugator activity can be considered as a better indicator of changes in fluency and affect. In an additional analysis, we took the activities of the corrugator muscle into account, along with the repetition status of presented information, to predict zygomaticus activity. Results revealed a significant positive effect of corrugator activity on zygomaticus activity, whereas the impact of repetition status was further reduced. These results suggest that the present zygomaticus activities are systematically associated with eyebrow frowning, indicating increased negative affect and mental effort when processing new (vs. repeated) information.^8^ Additional response time analyses revealed that muscle activities in all facial parts were systematically negatively related to judgmental speed, which is considered as an indicator of processing fluency in psychological literature. Similar to the above discussed EMG activities as a function of repetition status, the direction of the effects for the frontalis (+) and corrugator (+) muscles on response latencies appear well in line with previous research (Topolinski et al., 2009), while the direction of the effect for zygomaticus major (+) does not; again, this could be due to a rather quadratic relationship between valence and zygomaticus tension (Lang et al., 1993). Compared with the other two muscles, the corrugator activities appeared to be most strongly associated with the judgment times. Similarly, corrugator activities (compared with zygomaticus and frontalis activities) seemed to be most strongly influenced by information repetition; with the retention interval length significantly moderating the repetition effect only for the corrugator muscle. Looking at other EMG results in the fluency and affect literature, corrugator effects appear also most consistent. For example, Topolinski et al. (2009) found increased zygomaticus activities as well as reduced corrugator and frontalis activities when people read coherent (vs. incoherent) word triads. Topolinski and Strack (2015) observed increased corrugator activities associated with high surprising (vs. low surprising) statements, whereas zygomaticus and frontalis activities remained unaffected. Taken together, the pattern of findings described above suggests that the corrugator supercilii may be the most promising facial muscle for research on illusory truth as well as on fluency-related effects in general.

Integrating facial EMG activities in addition to the repetition status of information into the model to predict judgments of truth revealed that, besides repetition, decreased corrugator activities were significantly related to a higher probability of judging information as true. Furthermore, a mediation analysis indicated that corrugator activities partially mediated the effect of repetition status on truth judgments.^9^ The significant effects of repetition on facial EMG responses, and in consequence also the mediation effect related to corrugator activities, vanished after the longer time interval. These are findings which are consistent with earlier research suggesting a time-related reduction of the fluency experience including accompanying affective changes (Stump et al., 2022, Exp. 1) as well as the truth effect in general (Henderson et al., 2021). Analyses of response times strengthen this argumentation by showing that the negative effect of repetition on response latencies was reduced with a rising interval length. We propose that the time-related reductions of the repetition effects we observed may be due to reduced memory strengths after the longer (1 week vs. 10 min) retention interval; however, our pattern of results suggests that these are probably not related to explicit recollection of statements after a shorter interval. We explicitly informed our participants prior to the exposure phase that both true and false statements would be presented. If subjects explicitly remembered individual statements from the experiment, this should not be a valid cue for the evaluation of truth. In fact, earlier research findings by Arkes et al. (1989) even suggest that the truth effect is reduced when repeated statements are attributed to the experiment. In contrast, our results clearly show that the illusory truth effect is significantly more pronounced after the short interval compared with the long interval. Notably, reduced corrugator activities were systematically related to an increased probability of judging information as true after the short as well as long time interval (the retention interval length did not moderate this effect). A finding indicating that independently from the repetition status, the processing of certain statements may cause changes in affective states and mental effort (e.g., due to prior knowledge), which in turn impact subsequent truth judgments.

Previous research indicates that the relative processing fluency is decisive for the occurrence of a truth effect; Dechêne et al. (2009) observed an illusory truth effect only when disfluent and fluent stimuli were presented mixed. While we can reasonably assume that relatively higher processing fluency in the case of repeated (vs. new) information results in higher subjective truth, we know little about the extent to which experienced fluency or disfluency contributes to the effect. In view of the fact that repetition effects systematically decrease with increasing length of the retention interval, the temporal dynamics of muscle tensions for newly presented (disfluent) and repeated (fluent) statements can provide preliminary evidence. Specifically, the mean temporal EMG dynamics (Fig. 3) appear somewhat consistent for repeated statements after both retention intervals, whereas the temporal EMG dynamics for new claims seem to vary systematically as a function of retention interval length. This pattern indicates that the facial EMG activities are rather related to disfluency than fluency; future research is required to further decipher the role of experienced fluency and disfluency in the context of illusory truth. Looking at the temporal muscle dynamics, it furthermore seems like the corrugator activities peak later than the activities of zygomaticus and frontalis. Notably, all models for the prediction of truth judgments show that, in addition to the repetition status of the statements, only corrugator activities are systematically associated with the judgment (“true” vs. “false”). A potential cause for this pattern may be that, although EMG activities measured in all facial parts are related to encoding processes, corrugator activities (compared to frontalis and zygomaticus activities) are furthermore significantly related to truth judgments. This explanation remains speculative at this point but seems well in line with our additional analysis indicating that corrugator activities are systematically related to both (a) repetition and (b) the judgments of truth, as well as research by Noordewier and van Dijk (2018) indicating that after the onset of new valent stimuli, facial expressions are initially similar, whereas after some seconds of sensemaking, the facial responses begin to vary as a function of stimuli valence. One way to directly investigate the above line of argumentation would be to replicate our paradigm in future research, but this time not requiring subjects to complete an evaluative task (similar to Topolinski et al., 2009). That is, the participants' task would simply be to read new and repeated statements intermixed, allowing investigating the effects of information repetition on facial EMG activities independent of an evaluative setting.

Taken together, and to the best of our knowledge, no prior research has yet demonstrated a comparable physiological correlate of the illusory truth effect. Whereas the truth effect is often explained with repetition-based changes in fluency and affect, and fluency and affect give rise to largely overlapping facial responses, we should see these responses associated with changes in fluency and affect as a function of repetition. Indeed, this is exactly what we find, and this finding provides the first easy-to-detect physiological correlate of truth illusions. Furthermore, the current findings, revealing a systematic connection between facial muscle activities and subsequent truth judgments, hold significant implications from another perspective. These results indicate the potential for gaining valuable insights into the perceived credibility of disseminated information through the analyses of facial expressions. Such insights can be leveraged to validate and improve communication strategies across diverse domains, including politics and marketing. It is worth emphasizing the immense potential that our technological landscape already offers in this regard, which should be thoroughly examined, with front cameras becoming commonplace in a wide range of devices, alongside widely used applications requesting access to them. Moreover, the utilization of artificial intelligence (AI) can greatly facilitate the analysis of facial data.

We finally want to point out that our data were collected from young German adults, all of them university students. Thus, the generalizability of the effects to other cultural contexts, other age groups, or to groups with different educational backgrounds cannot be assessed based on the present data. Nonetheless, due to the fact that the truth effect has been demonstrated in many studies from various labs with different kinds of samples, we assume that this is a widespread phenomenon; therefore, we see no reason to assume that the fundamental cognitive and affective mechanisms investigated should be limited to the specific group examined in our study.

## Conclusions

With the present research, we demonstrate that the repetition of information results in specific facial reactions indicative of increased positive affect, decreased mental effort, and increased familiarity (i.e., relaxations of the musculus corrugator supercilii and frontalis), as well as an increased probability of judging information as true. Notably—even in case of novel statements—reduced corrugator activities were systematically related to an increased probability of judging information as true. Moreover, our results impressively highlight the significance of time on several levels: with increasing time between information presentations the effects of repetition are decreased for (a) the facial muscle responses indicating increased positive affect and decreased mental effort, (b) the response latencies for judgments, as well as (c) the probability of judging information as true.

## Data Availability

The used statement material, the data generated and analyzed during the current study is available in the heiDATA repository, 10.11588/data/2PKECX.

## Footnotes

1 In multilevel analyses, the level-2 sample size is often most relevant for power (Maas & Hox, 2005). Simulation studies by Maas and Hox (2005) suggest that for multilevel modeling, parameter estimations are of sufficient accuracy with at least 50 level-2 units (see, in addition, Paccagnella, 2011). The same minimum (50 level-2 units) is indicated for multilevel logistic regression models (Moineddin et al., 2007) to achieve valid estimates. With 75 level-2 units (participants) and 60 units per cluster (observations per judgment phase), the present study was well in line with all of these recommendations.

2 Because of light (LUX) sensor errors in certain trials, these had to be excluded. This concerned 1.8% of the trials in the first judgment phase and 1.4% of the trials in the second judgment phase.

3 Since convergence problems occurred when adding random intercepts for statements in several models, we decided to include only random intercepts for participants in our analyses.

4 We removed extreme values in the response time distribution prior to this analysis (outliers were defined as lying more than 3.0 interquartile ranges below the first or above the third quartile of the logarithmized response time distribution).

5 Response times are commonly used as a proxy for fluent processing (see e.g., Unkelbach & Rom, 2017; see in addition Reber et al., 2004).

6 Based on the suggestion of one reviewer, we also conducted the model using effect coded predictors, leaving the pattern of results unchanged (repetition status: *b* = −.191, *p* < .001; truth judgment: *b* = −.236, *p* < .001; repetition status x truth judgment: *b* = .037, *p* = .656).

7 While the main effects mentioned first were expected, the last finding seems somewhat surprising. Paradoxically, factually false statements were more likely to be judged as true; implying that, on average, the factually false statements that we used appeared to be more believable to our participants than the factually true statements.

8 Given the distance of the muscle areas involved (corrugator and zygomaticus), a so-called crosstalk seems very unlikely as a cause for the described association. [Crosstalk: When examining neighboring facial muscle areas, the electrodes are necessarily placed close to each other. This can lead to electrodes of a non-activated muscle area recording part of the activity of a neighboring activated muscle area and thus confounding the results; see also Farina et al., 2004].

9 In the context of statistical mediation analyses, it is important to point out that alternative mediators remain untested and other causal models could underlie the correlations investigated (for detailed discussion, see Fiedler et al., 2018). However, when considering the current fluency and affect literature combined with prior research findings and theories in the field of the truth effect, the causal model used can be reasonably preferred to others.
